# Inhibition of lysophosphatidic acid receptors 1 and 3 attenuates atherosclerosis development in LDL-receptor deficient mice

**DOI:** 10.1038/srep37585

**Published:** 2016-11-24

**Authors:** Eva Kritikou, Gijs H. M. van Puijvelde, Thomas van der Heijden, Peter J. van Santbrink, Maarten Swart, Frank H. Schaftenaar, Mara J. Kröner, Johan Kuiper, Ilze Bot

**Affiliations:** 1 Division of Biopharmaceutics, LACDR, Leiden University, The Netherlands

## Abstract

Lysophosphatidic acid (LPA) is a natural lysophospholipid present at high concentrations within lipid-rich atherosclerotic plaques. Upon local accumulation in the damaged vessels, LPA can act as a potent activator for various types of immune cells through its specific membrane receptors LPA_1/3._ LPA elicits chemotactic, pro-inflammatory and apoptotic effects that lead to atherosclerotic plaque progression. In this study we aimed to inhibit LPA signaling by means of LPA_1/3_ antagonism using the small molecule Ki16425. We show that LPA_1/3_ inhibition significantly impaired atherosclerosis progression. Treatment with Ki16425 also resulted in reduced CCL2 production and secretion, which led to less monocyte and neutrophil infiltration. Furthermore, we provide evidence that LPA_1/3_ blockade enhanced the percentage of non-inflammatory, Ly6C^low^ monocytes and CD4^+^ CD25^+^ FoxP3^+^ T-regulatory cells. Finally, we demonstrate that LPA_1/3_ antagonism mildly reduced plasma LDL cholesterol levels. Therefore, pharmacological inhibition of LPA_1/3_ receptors may prove a promising approach to diminish atherosclerosis development.

Atherosclerosis is a lipid-driven chronic inflammatory syndrome, accountable for the majority of acute cardiac episodes and comprising, at present, a principal cause of death in Western societies^1^. The disorder initiates upon damage of the arterial endothelium induced by high shear stress and excessive amounts of cholesterol in the form of low-density lipoproteins (LDL). LDL can accumulate within the subendothelial space, triggering the immune system to launch an inflammatory cascade^2^,^3^. Thereupon, circulating monocytes infiltrate and ingest modified LDL particles differentiating into macrophage foam cells, the main components of an atherosclerotic plaque. An additional influx of pro-atherogenic innate cells, such as neutrophils^4^ and mast cells^5^ follows, along with the rise of specific adaptive immune responses through presentation of lipid antigens by antigen presenting cells^6^. This process results in the infiltration of various subtypes of T-cells, the main one being CD4^+^ T-helper 1 (T_H1_) cells^7^. Evidently, an uncontrollable increase in the atherosclerotic plaque size or rupture of the plaque may lead to life-threatening clinical events.

Lysophosphatidic acid (LPA) is a bioactive glycerophospholipid found elevated in the circulation of patients with acute coronary syndrome^8^ and directly linked to hyperlipidemia^9^,^10^. The presence of LPA in the serum has been mainly associated with platelet activation^11^ but has also been described inside human atherosclerotic specimens^12^, as well as in the plaques of LDLr^−/−^ mice, with an increasing concentration rate upon disease progression^13^. Intraplaque LPA is enzymatically formed *in situ,* upon mild modification of the LDL molecule^14^ and elicits its effects via 9 different G-protein coupled receptors (LPA_1-6,_ GPR87, P2Y10 and GPR35) which have been classified into different subcategories based on their structural diversity and ligand specificity^15^,^16^. Of these receptors, LPA_1_, LPA_2_ and LPA_3_ are structurally similar and belong to the Endothelial Differentiation Gene (EDG) family of proteins^17^, with their general mode of action leading to gene regulation, chemokine secretion and cell survival^18^. Interestingly, LPA can activate mast cells^19^,^20^ and neutrophils^21^, as well as influence the migration of CD4^+^ T-helper cells^22^; all key cell types that are directly implicated in atherosclerosis. The impact of LPA on atherosclerosis has been thoroughly studied in the past, and reviewed in detail by Schober & Siess^23^, with its role extending from increased infiltration and activation of monocytes, to enhanced foam cell formation and elevated levels of endothelial permeability. Specifically, the chemotactic effects induced through LPA are linked to upregulation of inflammatory chemokines and adhesion molecules such as CCL2^24^, CXCL1^25^ and I-CAM-1^26^. The pro-atherosclerotic effects of LPA have been mainly tied to LPA_1_ and LPA_3,_ which were reported to increase atherosclerosis development in apoE^−/−^ mice^27^. Receptors LPA_1_ and LPA_3_ are widely expressed by immune cells^28^,^29^, as well as endothelial^30^ and vascular smooth muscle cells^31^, with LPA_3_ activation being involved in cell migration^32^ while LPA_1_ shows both migratory^33^ and apoptotic effects^34^.

In this study we aimed to examine the development of atherosclerosis upon pharmacological blockade of receptors LPA_1_ and LPA_3_ (LPA_1/3_), using the synthetic antagonistic compound Ki16425^35^.

## Results

### LPA_1/3_ inhibition reduces atherosclerotic plaque size

**Figure 1:**
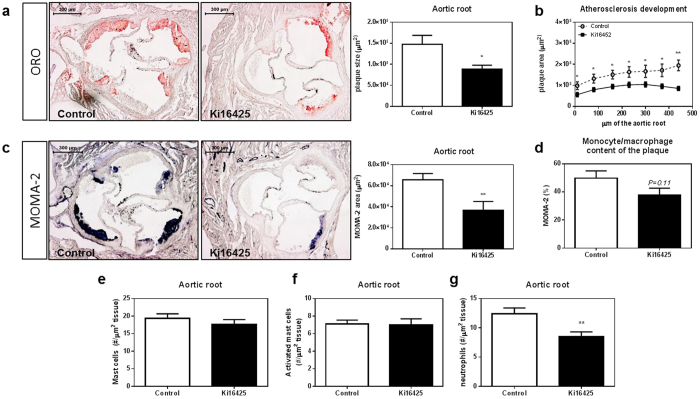
The LPA_1/3_ antagonist, Ki16425, reduces atherosclerosis development. **(a)** Atherosclerotic plaque size in the aortic root of the heart was determined by an Oil-Red-O staining on 10 μm sections; representative pictures are shown. Blockade of receptors LPA_1/3_ resulted in a 40% reduction in atherosclerosis. **(b)** The Ki16425 treated mice had significantly lower atherosclerotic plaque development throughout the entire three valve area of the aortic root. **(c)** Macrophage expression levels were measured using a MOMA-2 staining; LPA_1/3_ antagonism led to 45% less macrophage accumulation within the aortic root of the hearts. **(d)** The relative amount of macrophages in the atherosclerotic plaques was not significantly affected by the Ki16425 treatment. **(e)** Mast cell numbers (#) and **(f)** activation state, as well as **(g)** neutrophil numbers were manually quantified using a Naphthol AS-D chloro-acetate esterase staining; no difference was observed in the number or degranulation status of mast cells in the aortic root. Neutrophil numbers were found significantly reduced by 31% in the aortic root of the Ki16425 group as compared to the control. All values (n = 12/grp) are depicted as mean ± SEM (*P < 0.05, **P < 0.01).

To assess the effect of LPA_1/3_ inhibition on atherosclerosis, LDLr^−/−^ mice were injected intraperitoneally with either Ki16425 (5 mg/kg) or a vehicle-control for 6 weeks, (3x/week). Plaque size quantification, using an Oil-Red-O staining showed that mice treated with the LPA_1/3_ inhibitor had significantly smaller plaque size (−40%) compared to control mice (Fig. 1a, Ki16425: 89*10^3^ ± 9*10^3^ μm^2^ vs control: 147*10^3^ ± 21*10^3^ μm^2^, P = 0.023). In fact, plaque size was significantly lower in the treated group at each distance measured from the start of the three-valve area up to its end (Fig. 1b). A MOMA-2 staining was performed to evaluate the intra-plaque macrophage levels. The absolute macrophage content of the Ki16425 treated group was significantly lower (−45%) compared to the control (Fig. 1c, Ki16425: 36*10^3^ ± 8*10^3^ μm^2^ vs control: 65*10^3^ ± 6*10^3^ μm^2^, P = 0.006), whereas the relative amount (% macrophage levels of the plaque) was not significantly different (Fig. 1d, P = 0.11). Furthermore, the aortic root area was analyzed for mast cell and neutrophil content, since both immune cell types express LPA_1/3_ and are involved in atherosclerosis progression. No differences in the number or activation status of mast cells were detected between the two groups (Fig. 1e, P = 0.38 for mast cell # and Fig. 1f, P = 0.88 for activated mast cell #). However, a substantial reduction in the number of infiltrated neutrophils (−31%) upon LPA_1/3_ blockade was observed (Fig. 1g, Ki16425:8.5 ± 0.7 neutrophils/μm^2^ tissue vs. control: 12.4 ± 0.9 neutrophils/μm^2^ tissue, P = 0.004).

### Treatment with Ki16425 results in decreased serum total cholesterol levels

**Figure 2:**
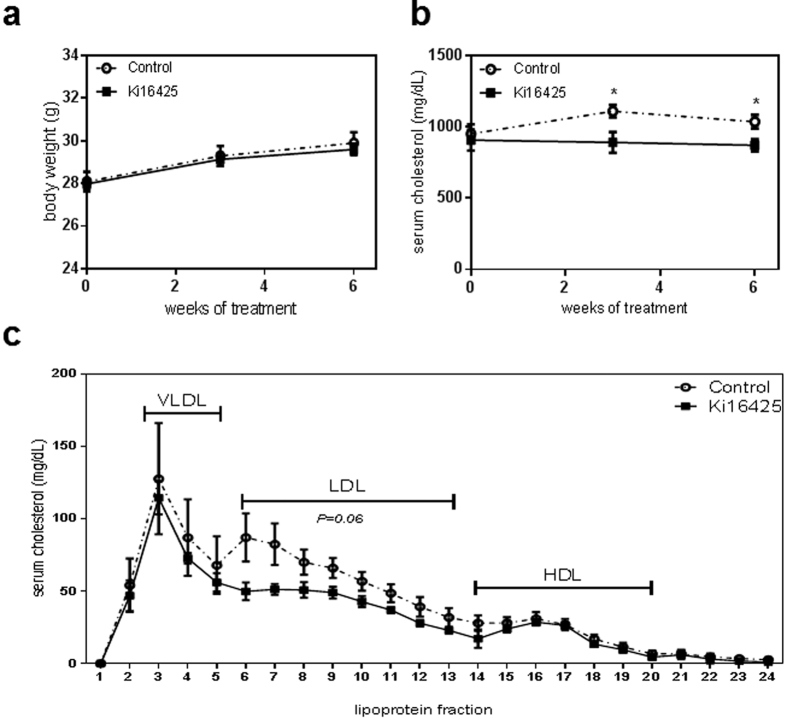
LPA_1/3_ inhibition reduces total cholesterol content in the serum. **(a)** The animal body weight showed no significant differences throughout 6 weeks of treatment between the groups. **(b)** Total serum cholesterol remained at significantly lower levels in the Ki16425 group compared to the control. **(c)** The Ki16425 treated animals presented a trend towards reduced LDL levels (P = 0.06). P-values are calculated by the fraction sum for each lipoprotein per group (n = 5/grp). All values are depicted as mean ± SEM (*P < 0.05).

LPA_1/3_ inhibition with Ki16425 did not alter body weight (Fig. 2a) and similarly, analysis of the liver weight or spleen weight at the endpoint of the study presented no change for the treated versus the control group (Supplementary Fig. 1a,b). Interestingly, total serum cholesterol levels in time upon LPA_1/3_ inhibition, was significantly lower compared to the control group (Fig. 2b, w3: −20%, P = 0.012; w6: −16%, P = 0.017). Further analysis of the serum lipoprotein content at the experimental endpoint showed a trend towards decreased LDL levels for the Ki16425 treated mice as compared to the control (Fig. 2c, P = 0.06).

### Pro-inflammatory CCL2 expression and secretion are reduced upon LPA_1/3_ inhibition

**Figure 3:**
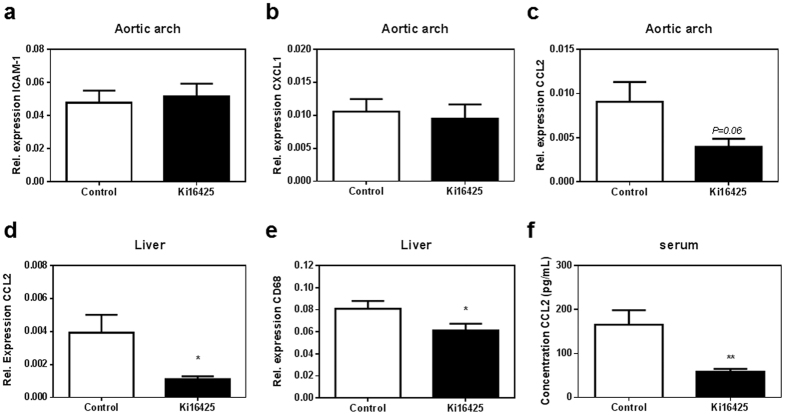
Treatment with Ki16425 decreases CCL2 chemokine expression and release. Gene profiling in the aortic arch displayed no difference between the two groups in **(a)** ICAM-1 or **(b)** CXCL1 chemokine expression levels. **(c)** CCL2 expression showed a slight reduction upon LPA_1/3_ antagonism. **(d)** Liver CCL2 expression was significantly lower in the Ki16425 treated animals compared to the control. **(e)** Macrophage CD68 gene expression in the liver was reduced for the Ki16425 mice compared to the controls. **(f)** CCL2 chemokine secretion was decreased up to 65% in the circulation of the Ki16425 group, compared to the control. Gene expression (n = 8/grp) is depicted as relative to two housekeeping genes (β-actin and Rpl27). Serum CCL2 concentration was measured using an ELISA assay (n = 13/grp). All values were calculated as mean ± SEM. (*P < 0.05, **P < 0.01).

Considering the lower macrophage and neutrophil content of the atherosclerotic plaque, we isolated mRNA from the aortic arch which was subsequently analyzed for the expression levels of different chemokines as well as endothelial adhesion molecules. The expression of ICAM-1, which is tightly linked to LPA^26^, did not present any differences between the control and Ki16425 group (Fig. 3a, P = 0.70). The same was observed for chemokine CXCL1 (Fig. 3b, P = 0.52). The pro-inflammatory chemokine CCL2 showed a trend towards reduction in the aortic arch of the Ki16425 treated mice (Fig. 3c, P = 0.067). Similarly, liver mRNA analysis displayed substantially lower CCL2 expression upon LPA_1/3_ antagonism (Fig. 3d, P = 0.035). In addition, gene expression of the macrophage marker CD68 was significantly reduced in the liver of animals treated with Ki16425 (Fig. 3e, P = 0.037). To establish whether the reduction in CCL2 expression results in diminished protein levels, CCL2 chemokine secretion in the circulation was determined and found considerably lower (−65%) upon LPA_1/3_ inhibition (Fig. 3f, Ki16425: 58.9 ± 6.4 pg/mL compared to 165.8 ± 32.8 pg/mL, P = 0.003).

### CCR2^+^ neutrophils and monocytes circulate at lower levels upon LPA_1/3_ antagonism

**Figure 4:**
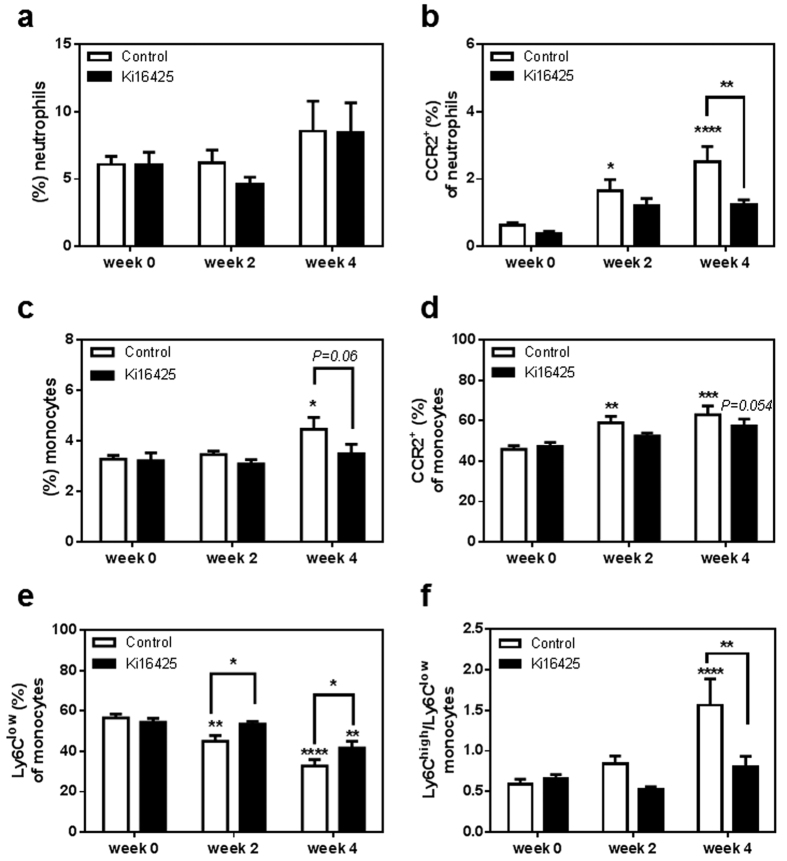
LPA_1/3_ inhibition retains circulating CCR2^+^ neutrophils and monocytes at low levels while increasing, Ly6C^low^ patrolling monocytes over time. (**a**) No difference was observed in the circulating neutrophil percentage between the two groups of animals. (**b**) CCR2^+^ expressing neutrophils were reduced after 4 weeks of LPA _1/3_ antagonism. (**c**) Circulating monocytes showed a slight reduction upon 4 weeks of Ki16425 treatment (**d**) CCR2^+^ monocytes remained at lower levels in the course of LPA_1/3_ inhibition. (**e**) Non-inflammatory monocytes appeared significantly higher at 2 and 4 weeks of LPA_1/3_ inhibition. (**f**) The ratio of inflammatory/non-inflammatory monocytes was increased in the control group compared to the treated. All values are calculated as mean ± SEM. (n = 12/grp, *P < 0.05, **P < 0.01, ***P < 0.001, **** P < 0.0001).

Since the reduced CCL2 levels could induce lower inflammatory cell infiltration, and therefore lower the plaque size, it was particularly intriguing to focus on immune cells that respond to this chemokine via its specific receptor, CCR2. For that reason two additional groups of LDLr^−/−^ mice were treated with either Ki16425 or vehicle-control for 6 weeks while on WTD. In the blood the overall percentage of circulating neutrophils, defined as Ly6G^+^ CD11b^+^/NK1.1^−^ cells, was not affected by the treatment (Fig. 4a). However, the relative amount of circulating CCR2^+^ neutrophils was significantly increased over time in the control group (Fig. 4b, control: w0 compared to w2, P = 0.031; w0 compared to w4, P = 0.0001), but was found reduced in the treated animals after 4 weeks of LPA_1/3_ inhibition (w4: Ki16425 compared to control, P = 0.0060).

Furthermore, the monocyte population in the circulation, defined as Ly6C^+^/CD11b^+^ Ly6G^−^/NK1.1^−^ cells was found to increase in the control group over 4 weeks of treatment (Fig. 4c, control: w0 compared to w4, P = 0.014). At week 4, the Ki16425 treated group showed a trend to reduced monocyte percentage when compared to the control (Fig. 4c, w4: Ki16425 compared to control, P = 0.066). Of this population, the CCR2^+^ monocytes, which comprise the inflammatory monocyte subset^36^ -alternatively defined as Ly6C^high^-increased greatly in the control group over time (Fig. 4d, control: w0 compared to w2, P = 0.004; w0 compared to w4, P = 0.0002), while the CCR2^+^ monocytes of the treated group were only slightly elevated (Ki16425: w0 compared to w2, P = 0.64; w0 compared to w4, P = 0.054).

On the contrary, Ly6C^low^-patrolling monocytes of the control mice showed a sharp decline over time (control: w0 compared to w2, P = 0.0039; w0 compared to w4, P < 0.0001), but upon Ki16425 treatment the decrease was only observed after 4 weeks and was less acute (Ki16425: w0 compared to w4, P = 0.0025). Therefore, in relation to the control group, LPA_1/3_ blocked animals presented significantly higher non-inflammatory circulating monocytes over time (Fig. 4e, Ki16425 compared to control: w2:P = 0.048; w4:P = 0.040). Hence LPA_1/3_ inhibition retained the inflammatory versus non-inflammatory responses at lower levels as shown by the relative difference of inflammatory versus the non-inflammatory monocytes per group (Fig. 4f, Ki16425 w4 compared to control w4: P = 0.003).

### LPA_1/3_ inhibition increases circulating anti-inflammatory CD4^+^ T-regulatory cells, while decreasing T helper-1 cells

**Figure 5:**
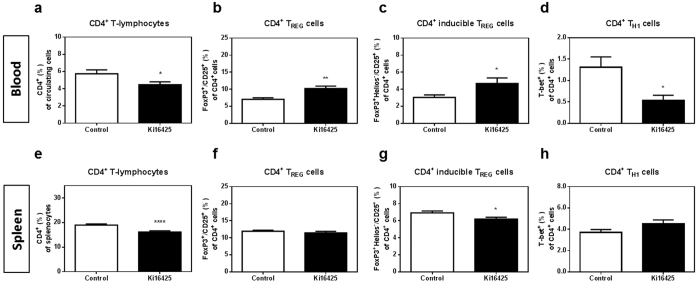
Systemic treatment with Ki16425 for 6 weeks reduces CD4^+^ T-cells in the blood and spleen, with a potency to increase anti-inflammatory T_REG_ cells. (**a**) Upon 6 weeks of treatment, LPA_1/3_ antagonism reduced the percentage of CD4^+^ T-cells in the blood of LPA_1/3_ blocked mice, as compared to the control. (**b**) Anti-inflammatory CD4^+^ T_REG_ and (**c**) inducible FoxP3^+^ Helios^−^ T_REG_ cells were detected at higher levels in contrast to (**d**) lower inflammatory T_H1_ cells in the blood of the Ki16425 treated mice. (**e**) In the spleen of Ki16425 treated animals CD4^+^ T-cells appeared significantly lower. (**f**) No difference was observed within the overall CD4^+^ T_REG_ cells. (**g**) The inducible T_REG_ cells were found substantially decreased. (**h**) No significant difference was detected in the CD4^+^ T_H1_ population. All values are calculated as mean ± SEM. (n = 12/grp, *P < 0.05,**P < 0.01, ****P < 0.0001).

Flow cytometric analysis of the white blood cell population showed a significant reduction in the circulating CD4^+^ T-cell percentage after LPA_1/3_ inhibition (Fig. 5a, P = 0.036). However, within the CD4^+^ T-cell population, anti-inflammatory FoxP3^+^/CD25^+^/CD4^+^ T_REG_ percentage was found significantly increased (Fig. 5b, Ki16425: 10.24 ± 0.71% versus control: 7.06 ± 0.41%, P = 0.0013). Among the CD4^+^ T_REG_ population, FoxP3^+^ Helios^−^/CD25^+^ cells, defined as inducible (i) T_REG_ and generated in secondary lymphoid organs^37^,^38^, increased significantly in the Ki16425-treated group (Fig. 5c, P = 0.038). The percentage of pro-inflammatory CD4^+^ T_H1_ cells in the blood was reduced upon LPA_1/3_ inhibition (Fig. 5d, P = 0.011). Also in the spleen, CD4^+^ T-cells were reduced in the Ki16425 treated mice (Fig. 5e, P < 0.0001), with no difference observed however in the overall FoxP3^+^/CD25^+^/CD4^+^ T_REG_ population (Fig. 5f, P = 0.39). Nonetheless, LPA_1/3_ inhibition resulted in a significant reduction in the inducible CD4^+^ T_REG_ cells (Fig. 5g, P = 0.025). No significant differences in T_H1_ cells were observed in this compartment (Fig. 5h, P = 0.09).

In addition, no difference was observed within the CD8^+^ population present in the blood or spleen of the two groups (Supplementary Fig. 2a,b). Likewise, MHC-II^+^ CD11c^high^ dendritic cells (DCs) were not affected by the treatment (Supplementary Fig. 3c).

### T-cell content of the atherosclerotic plaque is not altered by LPA_1/3_ inhibition

**Figure 6:**
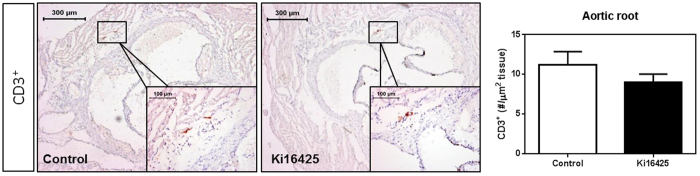
Total CD3^+^ T-cell numbers in the aortic root show no difference between the control and Ki16425-treated groups. CD3^+^ cells in the aortic root of the hearts were determined upon manual quantification. No difference was observed in the CD3^+^ expressing cells of the aortic root upon LPA_1/3_ inhibition. All values (n = 12/grp) are depicted as mean ± SEM.

Considering that the systemic differences in the CD4^+^ T-cells upon LPA_1/3_ blockade may directly influence the T-cell content inside the atherosclerotic plaque, a CD3^+^ immunohistochemical staining was performed in the aortic root. However, no significant differences were observed upon manual quantification of the CD3^+^ cells in the three-valve area of both groups (Fig. 6, P = 0.43).

## Discussion

In this study we show that pharmacological inhibition of LPA_1/3_ receptors by Ki16425 reduced atherosclerotic plaque development by 40%. Specifically, we have found that LPA_1/3_ antagonism significantly attenuated the macrophage and neutrophil content of the plaque. Furthermore, a mild down-regulation of total serum cholesterol was detected, which may have contributed to the decreased atherosclerotic plaque size. We observed a systemic reduction in the expression and secretion of chemokine CCL2, which could have resulted in the detected CCR2^+^ monocyte and neutrophil decline in the circulation. This decline in CCR2^+^ cells in the blood seems consistent with the reduction in the absolute amount of macrophages and neutrophils inside the atherosclerotic plaques of the Ki16425 mice. Upon lower levels of circulating CCL2, less CCR2^+^ monocytes and neutrophils are infiltrating the atherosclerotic site and subsequently there is a reduction in the absolute number of neutrophils and macrophages observed inside the plaque. As the atherosclerotic plaque at this specific time-point consists primarily of macrophages, the absolute amount actually reflected the reduction in plaque size, whereas the relative macrophage content did not differ. This response is in line with previous evidence supporting that LPA elicits its effects through the release of CCL2 by its target cells^19^. Unlike reports that link the action of LPA to endothelial cell activation^12^,^26^,^39^, in this investigation we did not observe any differences in the aortic ICAM-1 or CXCL1 expression levels. It is important to mention however that in a previously published study by Zhou *et al*., the atherosclerotic effects of LPA were mainly associated with endothelial activation through chemokine CXCL1^25^ and not with the CCL2-CCR2 axis. Furthermore, in their study no effect was observed on cholesterol metabolism, whereas we detected a reduction in LDL cholesterol levels. Considering that the above experiments were performed in apoE^−/−^ mice, it is possible that strain specific effects are responsible for the different mechanism observed^40^. In the past, numerous studies have examined the differences between the LDLr^−/−^ and apoE^−/−^ models. The leading distinction between the two strains is that LDLr^−/−^ mice do not develop atherosclerosis unless placed on a high-cholesterol diet^42^, whereas apoE^−/−^ mice have basal plaque formation even on chow diet^41^. Thereupon, apoE^−/−^ mice develop higher plasma cholesterol levels and more pronounced plaques upon WTD, while showing lower plaque T-cell numbers^43^. In addition, apoE^−/−^ mice, unlike the LDLr^−/−^, exhibit impaired formation and efflux of HDL particles, an effect which is highly distinct from the human case^44^. With the above in mind, we considered LDLr^−/−^ as a model that does not show atherosclerosis-related systemic effects prior to WTD. Importantly the presence of apoE can affect monocyte formation and accumulation in the plaque^45^, which could account for the main difference between the two models in regard to the role of LPA_1/3_ receptors. In addition, apoE^−/−^ and LDLr^−/−^ mice have also proven to differ in the ABCA1-mediated cholesterol transportation pathway^46^,^47^. This may explain the reason why LDLr^−/−^ mice show differences in cholesterol regulation upon LPA_1/3_ inhibition, an effect which was absent in apoE^−/−^ mice. Nevertheless, in both experimental sets LPA_1/3_ inhibition substantially reduced plaque development to a similar extent despite the fact that the apoE^−/−^ mice were treated with Ki16425 daily for 3 months while in our study LDLr^−/−^ mice were injected 3x/week for a 6 week period.

Furthermore, in our study, the patrolling Ly6C^low^ population was increased already after two weeks of Ki16425 treatment, suggesting an additional anti-inflammatory effect. This anti-inflammatory subtype of mouse monocytes expressing the Ly6C protein at low levels, has been previously reported to play a crucial role in the control of atherosclerosis development since LDLr^−/−^ mice that lack this subtype showed increased plaque formation^48^. Therefore, the anti-inflammatory effects of patrolling monocytes detected upon LPA_1/3_ antagonism may have contributed to the reduction in atherosclerosis development. Together, it seems that Ki16425 carries out its effects primarily by regulating the immune response, since also the CCR2^+^ monocytes and neutrophils are circulating at lower levels compared to the control already at the second week of treatment. This does not exclude the fact that cholesterol lowering mechanisms may have been partly responsible for the reduced disease progression.

In the past, LPA has also been reported to affect the proliferation of T-lymphocytes^49^. Here we observe that upon LPA_1/3_ antagonism, CD4^+^ T-cells are substantially diminished, which may have been due to a reduction in LPA induced mitogenesis^18^. Furthermore, LPA can promote the migration of CD4^+^ T-cells^33^, but not of CD8^+^ T-cells^50^. This is in line with our observations on CD4^+^ T-cells being markedly reduced upon LPA_1/3_ inhibition, while no effects were detectable on the CD8^+^ T-cell population in either the blood or spleen. Similarly, no differences were found in the dendritic cell population; however, the effects of LPA on DCs seem to depend on their state. For example, mouse DCs were previously reported to migrate towards LPA upon LPA_3_ activation, yet for that to take place they had to be in an immature state^51^. As mentioned above, CD4^+^ T-cells can migrate in response to LPA, but this action was previously shown to depend on the expression of LPA_1_ and LPA_2_. Specifically, these two receptors are considered to have an inverse impact on T-cell migration, with LPA_1_ enhancing it while LPA_2_ hinders it, depending though also on whether the cells are in a naive or activated state^50^. This fact further illustrates the complexity behind LPA signaling and how different receptor signaling pathways can have entirely opposing actions.

Notably, while CD4^+^ T-cell levels were reduced upon Ki16425 treatment, the anti-inflammatory T_REG_ cells were strongly enhanced. To our knowledge, there is thus far no evidence describing the relationship between LPA_1/3_ inhibition and T_REG_ induction. The observed effects indicate that LPA_1/3_ inhibition can skew the adaptive immune system towards an anti-inflammatory response as demonstrated by the overall lower CD4^+^ levels, with the pro-inflammatory T_H1_ subset found relatively decreased in the blood while the athero-protective^52^ CD4^+^ T_REG_ cells were circulating at increased levels.

However, the differences on the CD4^+^ population in the blood and spleen did not reflect the T-cell numbers inside the plaques of the treated mice. This suggests that the reduced atherosclerotic plaque size observed upon LPA_1/3_ antagonism was not mediated through a difference in local T cell responses, but was rather elicited via a systemic anti-inflammatory effect of the Ki16425 treatment. Moreover, we found no difference on the mast cell counts in the atherosclerotic plaques of the treated mice, despite previous evidence that mast cells respond to LPA through LPA_1/3_ receptors^53^. Recent studies have also implicated additional LPA receptors present on the mast cell surface, which may induce mast cell activation in an LPA_1/3_ independent fashion^54^. Furthermore, mast cell effects mediated by LPA could also depend on the stage of atherosclerosis. For instance, our group has previously reported that mast cell activation through LPA leads to advanced plaque destabilization^19^, while in this study we aimed our attention in early plaque development.

In conclusion, LPA_1/3_ receptor inhibition through Ki16425 induced systemic anti-inflammatory responses via a reduction in CCL2-CCR2 signaling, an enhancement of anti-inflammatory innate as well as adaptive immune responses and a decrease in plasma cholesterol levels, collectively resulting in the reduction of the atherosclerotic burden. The anti-inflammatory immune reaction of Ki16425 may span even outside atherosclerosis and into other diseases, which are characterized by excessive inflammation. For instance, Ki16425 has been found to reduce inflammation in experimental models for obesity^55^, rheumatoid arthritis^56^, and lung fibrosis^57^. Thus, the importance of singularly targeting the LPA_1/3_ receptors via pharmacological inhibition may grant anti-inflammatory effects without completely shutting down the immune response. At the same time it retains possible positive effects that LPA may evoke through its additional receptors in other physiological processes inside the body. Therefore, LPA_1/3_ inhibition is an interesting therapy with multiple beneficial effects that can be employed in a broad spectrum of diseases, among which atherosclerosis.

## Methods

### Animals

LDLr^−/−^mice were initially obtained from the Jackson Laboratories and bred at the Leiden University animal facility with water and food supply *ad libitum*. All animal work was approved by the Leiden University Animal Ethics committee and performed according to the guidelines established by the Dutch government and European Union.

### Atherosclerosis

Atherosclerosis was induced in male LDLr^−/−^ mice (10–11 weeks old) by feeding a Western type diet (WTD) (0.25% cholesterol, 15% cocoa butter; Special Diet Services, Essex, UK) for 2 weeks prior and throughout the course of the treatment. Subsequently, 12–15 mice per group were randomized based on age, weight and/or cholesterol levels and injected intraperitoneally 3 times a week with either a vehicle control or 5 mg/kg of the LPA_1/3_ antagonist, Ki16425 (Cayman Chemical); the injections were performed for 6 weeks. All mice were monitored for body weight changes every week throughout the treatment period; at the experimental endpoint, weight was additionally determined in organs such as the spleen and liver.

### Immunohistochemistry

The mice were anaesthetized with a mix of ketamine (100 mg/ml), sedazine (25 mg/ml) and atropine (0.5 mg/ml) and perfused with PBS through heart puncture in the ventricles. The hearts were dissected below the atria and sectioned perpendicularly to the axis of the aorta, starting within the heart and in the direction towards the aortic arch. Upon aortic root identification by the appearance of the aortic valve leaflets, 10 μm sections were collected and mounted on gelatin-coated slides. Mean plaque area (in μm^2^) was calculated for six sequential Oil-Red-O stained sections in distal direction, starting at the point where all three aortic valve leaflets first appeared. Plaque macrophages were stained using a MOMA-2 antibody at a 1:1000 concentration (rat IgG2b, Serotec Ltd.). For the MOMA-2 levels in the plaques, three subsequent sections displaying the highest plaque content per mouse were analyzed. Mast cells and neutrophils were visualized by staining with a Naphthol AS-D chloro-acetate esterase kit (Sigma Aldrich) and counted manually. A mast cell was considered resting when all granules were maintained inside the cell, while mast cells were assessed as activated when granules were deposited in the tissue surrounding the mast cell. Neutrophils were identified as round cells with a characteristic lobular nucleus and pink granular cytoplasm. T cell numbers were determined following a CD3 staining at a 1:150 concentration (clone S7P, ThermoScientific). All microscopic analyses were performed on a Leica DM-RE microscope and ORO as well as MOMA-2 quantifications were carried out using Leica QWin software (Leica, Imaging Systems, UK) and through blinded independent analysis.

### RT-PCR

Isolation of mRNA was performed on the liver and aortic arch of 8 mice/group, based on the guanidium isothiocyanate method^58^. Subsequently, the M-MuLV reverse transcriptase (RevertAid, Leon-Roth) was used for the reverse transcription. The quantitative analysis of specific gene expression was executed on a 7500 Fast-real time PCR system (Applied Biosystems, Foster City, CA) using SYBR Green Technology. The relative expression was determined based on two housekeeping genes; β-actin and ribosomal protein L27 (Rpl27). The complete primer list is included in the Supplementary Table S1.

### Serum analysis

Throughout the study, tail blood was collected by a tail-vein cut; serum was obtained by centrifugation at 8.000 rpm for 10 minutes. Total cholesterol levels in the serum were measured at week 0, 3 and 6 of the treatment, by an enzymatic colorimetric assay and in comparison to an internal Precipath control (standard serum provided by Roche Diagnostics). At the end of the experiment, cholesterol fraction separation for lipoprotein particle analysis was obtained after processing 30 μL serum/mouse through a Superose 6 column (Smart System, Pharmacia). Subsequent measurement of cholesterol levels in the retrieved fractions was performed as described above. CCL2 levels in the serum of 13 mice/group were analyzed by ELISA (BD Biosciences) according to the manufacturer’s protocol.

### Flow cytometry

Blood was collected as described above; Red blood cells were lysed using an erythrocyte lysis buffer (0.1 mM EDTA, 10 mM NaHCO_3_, 1 mM NH_4_Cl, pH = 7.2). Subsequently, white blood cells were stained with the relevant antibodies for flow cytometry analysis (Supplementary Table S2). Spleen tissue was harvested from all mice and processed through a 70 μm cell strainer to acquire single cell suspensions. Subsequently, cells underwent lysis once, for erythrocyte disposal, and were stained for flow cytometry. In approximation, 200.000 cells per sample were stained with antibodies against extracellular proteins at a concentration of 0.1 μg/sample for 30 minutes. For the detection of intracellular antibodies all cells were fixated, permeabilized using the transcription factor kit (Ebioscience) and stained at a concentration of 0.3 μg/sample. All flow cytometry experiments were executed on a FACS Canto II and data were analyzed using FlowJo software.

### Statistics

All data were analyzed using the GraphPad Prism software and presented as mean ± SEM. A 2-tailed Student’s t-test was used to compare individual groups. Non-Gaussian distributed data were analyzed using a 2-tailed Mann-Whitney U test. For the analysis of two or more variables a two-way ANOVA was used with the Bonferroni post-test for multiple comparisons. The probability (alpha) for all tests was set to 0.05 with values lower than this considered significant (P < 0.05).

## Additional Information

**How to cite this article**: Kritikou, E. *et al*. Inhibition of lysophosphatidic acid receptors 1 and 3 attenuates atherosclerosis development in LDL-receptor deficient mice. *Sci. Rep.* **6**, 37585; doi: 10.1038/srep37585 (2016).

**Publisher’s note:** Springer Nature remains neutral with regard to jurisdictional claims in published maps and institutional affiliations.

## Supplementary Material

Supplementary Information
