# Supplementary material for: Inhibition of lysophosphatidic acid receptors 1 and 3 attenuates atherosclerosis development in LDL-receptor deficient mice
Source: Sci Rep. 2016 Nov 24;6:37585. doi: 10.1038/srep37585 (PMC5121611; doi:10.1038/srep37585)
Supplement: Supplementary Information [file srep37585-s1.pdf]

Supplementary Information

Inhibition of lysophosphatidic acid receptors 1 and 3 attenuates atherosclerosis development in LDL-receptor deficient mice

Kritikou E., van Puijvelde G.H.M., van der Heijden T., van Santbrink P.J., Swart M., Schaftenaar F.H., Kröner M.J., Kuiper J., Bot I.

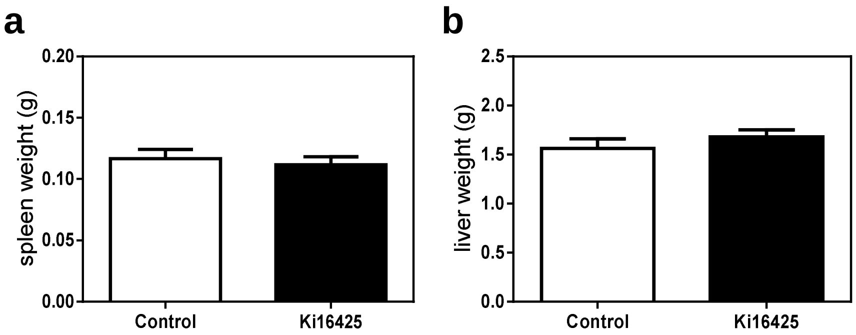

Figure 1: Treatment with Ki16425 does not affect the spleen or liver weight of mice. (a) The spleen weight between the control and Ki16425 treated group showed no significant difference (P=0.63) at the end of the study. **(b)** Similarly the liver weight between the control and Ki16425 groups was not substantially different (P=0.38). All values (n=13/grp; spleen and n=6/grp; liver) are depicted as mean±SEM.

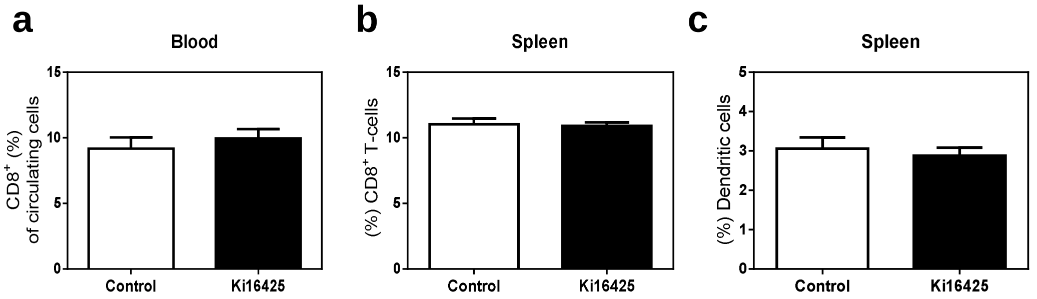

Figure 2. $LPA_{1/3}$ inhibition did not affect the amount of $CD8^+$ T-lymphocytes or dendritic cells. The amount of $CD8^+$ T-cells in the **(a)** circulation and **(b)** spleen was not affected by treatment with Ki16425. (P=0.50) and (P=0.78). **(c)** The overall percentage of dendritic cells present in the spleen showed no differences among the control and treated mice (P=0.60). (All values (n=12/grp) are depicted as mean±SEM.

Gene	*Forward primer (5'-3')*	*Reverse primer (3'-5')*
CCL2	GCATCT GCCCTAAGGTCT TCA	TTCACTGTCACACTGGTCACTCCTA
CD68	TGCCTGACAAGGGACACTTCGGG	GCGGGTGATGCAGAAGGCGATG
CXCL1	TTGACCCTGAAGCTCCCTTG	AGGTGCCATCAGAGCAGTC
I-CAM 1	GTCCGCTTCCGCTACCATCA	GGTCCTTGCCTACTTGCTGCC
Rpl27	CGCCAAGCGATCCAAGATCAAGTCC	AGCTGGGTCCCTGAACACATCCTTG
β-actin	AACCGTGAAAAGATGACCCAGAT	CACAGCCTGGATGGCTACGTA

Supplementary Table 1. qPCR gene primer sequences: All gene expression analysis was performed using two housekeeping genes (β-actin and Rpl27). Abbreviations: Chemokine C-C motif ligand 2 (CCL2); cluster of differentiation 68 (CD68); chemokine C-X-C motif ligand 1 (CXCL1); Intercellular adhesion molecule-1 (ICAM-1); 60s ribosomal protein ligand 27 (Rpl27).

**Antibody**	**Fluorochrome**	**Clone**	**Company**
CD11b	eFluor 450	M1/70	Ebioscience
Ly6C	PercP Cy5.5	HK1.4	Ebioscience
Ly6G	PE	1A8	BD Biosciences
CCR2	APC	#475301	R&D Systems
CD4	PercP	Rm4-5	BD Biosciences
CD25	FITC	PC61.5	Ebioscience
NK1.1	FITC	PK136	Ebioscience
CD8a	PercP	53-6.7	BD Biosciences
MHC-II	eFluor 450	AF6-120.1	Ebioscience
CD11c	APC	N418	Ebioscience
FoxP3	*eFluor 450*	*FJK-16s*	Ebioscience
T-bet	*Alexa -Fluor 660*	*eBio4B10*	Ebioscience
Helios	*Alexa-Fluor 647*	*22F6*	Ebioscience

Supplementary Table 2. List of extracellular and *intracellular* antibodies used.
